# Identification and functional characterization of multiple inositol polyphosphate phosphatase1 (Minpp1) isoform-2 in exosomes with potential to modulate tumor microenvironment

**DOI:** 10.1371/journal.pone.0264451

**Published:** 2022-03-02

**Authors:** Mohd Zubair, Rabab Hamzah, Robert Griffin, Nawab Ali

**Affiliations:** 1 Department of Biology, University of Arkansas at Little Rock, Little Rock, AR, United States of America; 2 Department of Radiation Oncology, University of Arkansas for Medical Sciences, Little Rock, AR, United States of America; 3 Center for Integrative Nanotechnology Sciences, University of Arkansas at Little Rock, Little Rock, AR, United States of America; MAHSA University, Malaysia, MALAYSIA

## Abstract

Inositol polyphosphates (InsPs) play key signaling roles in diverse cellular functions, including calcium homeostasis, cell survival and death. Multiple inositol polyphosphate phosphatase 1 (Minpp1) affects the cellular levels of InsPs and cell functions. The Minpp1 is an endoplasmic reticulum (ER) resident but localizes away from its cytosolic InsPs substrates. The current study examines the heterogeneity of Minpp1 and the potential physiologic impact of Minpp1 isoforms, distinct motifs, subcellular distribution, and enzymatic potential. The NCBI database was used to analyze the proteome diversity of Minpp1 using bioinformatics tools. The analysis revealed that translation of three different Minpp1 *variants* resulted in three isoforms of Minpp1 of varying molecular weights. A link between the *minpp1 variant-2* gene and ER-stress, using real-time PCR, suggests a functional similarity between *minpp1 variant-1 and variant-2*. A detailed study on motifs revealed Minpp1 isoform-2 is the only other isoform, besides isoform-1, that carries a phosphatase motif for InsPs hydrolysis but no ER-retention signal. The confocal microscopy revealed that the Minpp1 isoform-1 predominantly localized near the nucleus with a GRP-78 ER marker, while Minpp1 isoform-2 was scattered more towards the cell periphery where it co-localizes with the plasma membrane-destined multivesicular bodies biomarker CD63. MCF-7 cells were used to establish that Minpp1 isoform-2 is secreted into exosomes. Brefeldin A treatment resulted in overexpression of the exosome-associated Minpp1 isoform-2, suggesting its secretion via an unconventional route involving endocytic-generated vesicles and a link to ER stress. Results further demonstrated that the exosome-associated Minpp1 isoform-2 was enzymatically active. Overall, the data support the possibility that an extracellular form of enzymatically active Minpp1 isoform-2 mitigates any anti-proliferative actions of extracellular InsPs, thereby also impacting the makeup of the tumor microenvironment.

## Introduction

Inositol polyphosphates (InsPs) are naturally occurring compounds widely distributed from unicellular to multicellular organisms. Higher organisms are well recognized for their critical signaling role in maintaining cellular calcium homeostasis [1]. Among the InsPs, Inositol (1,2,3,4,5,6) hexakisphosphate (InsP_6_), also known as phytic acid, is the most abundant in nature, regulates critical cell biological processes in both plants and animals [2–6]. InsP_6_, also a substrate, produces highly energetic pyrophosphates (PP-InsPs: InsP_7_ and InsP_8_), implicated in various cellular processes such as apoptosis [7], vesicular trafficking [8, 9], ribosome biogenesis [10], aging [11] and energy metabolism [12]. Overall, InsP_6_ and its derivatives have been reported as anti-proliferative agents that counteract tumorigenic processes reverting a cell to normalcy [13, 14]. Indeed, the homeostasis of InsPs is maintained by a complex metabolic network of InsPs regulated by InsPs specific kinases and phosphatases [15, 16].

Multiple inositol polyphosphate phosphatase1 (Minpp1), a member of the histidine phosphatase family [17], removes the 3-phosphate from higher InsPs including Ins(1,3,4,5)P_4_, Ins(1,3,4,5,6)P_5_, InsP_6_, InsP_7_ and InsP_8_ converting them to lower InsPs [18, 19]. However, after Minpp1 was shown to be compartmentalized in the lumen of ER [19] without any access to its substrate (predominantly cytosolic InsPs), researchers explored other possible physiological functions of Minpp1. In this regard, attempts have been made to find heterogeneity in its structure and function based on gene variants and possible protein isoforms [20–22]. Recent studies from various research groups [17, 23–28], including studies from this lab [22], have suggested relatedness, divergence, and complexity adopted in Minpp1 structure through evolution. More recently, Minpp1’s extra-ER location [28–31] in various studies supports Minpp1’s isoform multiplicity (IM) [32–36]. However, the identity of these variants/isoforms has not yet been characterized biochemically in mammalian cells.

ER is an essential organelle in protein folding and their secretion to various cellular destinations. In a conventional protein secretory pathway, inherent signal sequences, such as KDEL, KKXX, etc., sort proteins into destined locations. Nonetheless, not all proteins are secreted through the conventional secretory pathway. Leaderless or signal-peptide-containing proteins follow an unconventional protein secretory (UPS) pathway involving endocytic vesicles, secretory lysosomes and multivesicular bodies/endosomes (MVB/MVEs) to the plasma membrane (PM) and extracellular space [37]. Small cytosolic gaps between associating membranes such as ER-endosome contact sites [38] provide a non-vesicular exchange of lipid-bound proteins [39], metabolites, and calcium ions [40] to heterogeneous intraluminal vesicles (ILVs) of MVBs [41–44]. However, the exact mechanism of protein and lipid sorting into ILVs destined for extracellular space, enclosed in extracellular vesicles (EVs), is still unknown. These EVs comprise a heterogeneous mixture of exosomes and ectosomes [46–48], ranging from 40-1000nm in diameter in their most current classification. Of EVs, exosomes ranging from 40nm to 200nm in diameter are the only vesicles of endocytic-origin [45] enclosing a diverse mixture of RNAs, miRNA, proteins, lipids, and glycans [46] implicated in various immune modulations [47] and disease states [48–50].

Since Minpp1 is an ER-restricted enzyme due to the presence of an ER retention signal (KDEL/SDEL) at its C-terminus, its presence in extra-ER compartments seems improbable. However, a previous study found a Minpp1 enzymatic activity associated with lysosomes and extracellular cell culture media [51]. Suggesting a possibility of heterogeneity in Minpp1 structure and function and the presence of a Minpp1 isoform in extra-ER location. Furthermore, if enzymatically active, this isoform would have physiological consequences in neutralizing the anti-proliferative actions of extracellular InsPs, thus modulating the tumor microenvironment [51].

This study explored Minpp1 heterogeneity and identified a Minpp1 isoform-2 in extracellular vesicles (exosomes) isolated from a human breast cancer cell line, MCF-7. Initially, relative expression profiling of *minpp1* variants was performed during cellular stress conditions and then correlated with Minpp1 isoform-2 secretion in exosomes to study its prospective secretory route. To further correlate the role of Minpp1 isoform-2 in dephosphorylation of extracellular InsP_6_, the enzymatic potential of the extracellularly secreted Minpp1 isoform-2 in exosomes was analyzed. The results suggest a potential role for exosome-associated Minpp1 isoform-2 in promoting tumor cell growth by preventing anti-proliferative actions of InsP_6_.

## Material and methods

### Materials

Phosphate Buffer Saline (PBS, pH 7.4) used in all experiments were Ca^+2^ and Mg^+2^ free. TriTrack DNA Loading Dye (6x), Tunicamycin and CHAPS were purchased from Thermo Fisher Scientific (Waltham, MA). Sequencing grade trypsin (0.25% trypsin-EDTA) was purchased from Caisson Labs (Smithfield, UT). D-myo-inositol hexakisphosphate (InsP_6_, dodecasodium salt) and Brefeldin A (BFA) were purchased from Sigma Aldrich (St. Louis, MO). D-myo-inositol 1,3,4,5-tetrakisphosphate (InsP_4_) was procured from Echelon Biosciences (Salt Lake City, Utah). Human Ins(1,4,5)P_3_ ELISA kit (MBS702115) was obtained from My BioSource (San Diego, CA). Transfection reagent (TransIT-2020) was purchased from Mirius Bio (Madison, WI). Human *minpp1 variant-2* GFP tagged (HG29732-ACG) and *minpp1 variant-1* OFPSpark/RFP tagged (HG16673-ACR) cDNA clones were obtained from Sino Biological (Wayne, PA). Mouse anti-CD63 mAb (H5C6), mouse anti-lamp2 mAb (H4B4), and mouse anti-CD9 mAb (602.29.cl.11) were obtained from Developmental Studies Hybridoma Bank (DSHB) (Iowa City, IA). Mouse anti-GFP tagged mAb was obtained from ProteinTech Group (Rosemont, IL). Rabbit anti-Minpp1 polyclonal antibody was procured from Fabgennix Inc. (Frisco, Texas). Mouse anti-Minpp1 mAb (A-8), mouse anti-CD63, and HRP conjugated anti-mouse binding protein (m-IgGk BP-HRP) were obtained from Santa Cruz Biotechnology (Dallas, Texas). Peroxidase conjugated IgG mouse anti-rabbit light chain specific monoclonal antibody was obtained from Jackson Immuno Research (Philadelphia, PA). Formvar carbon-coated grids 300 mesh were purchased from Electron Microscopy Sciences (Hatfield, PA). All other reagents were purchased from Sigma-Aldrich (St. Louis, MO).

### Cell culture

HeLa (CCL-2), MCF-7 (HTB-22) and, MDA-MB231 (HTB-26) cells were obtained from American Type Culture Collection (ATCC) (Manassas, VA) and maintained in DMEM supplemented with 10% fetal bovine serum, Bio-techne (Minneapolis, MN) and 1% Penicillin-Streptomycin, Thermo Fisher Scientific (Waltham, MA). MCF-10A (ATCC-CRL-10317) cells were a kind gift from Dr. Chambers at the University of Arkansas for Medical Sciences (UAMS) and were maintained in Mammary Epithelial Basal Cell Medium, Promo Cell (Heidelberg, Germany) supplemented with Mammary Epithelial Cell Growth Medium Supplement Pack, Promo Cell (Heildelberg, Germany) in addition to 100μg/mL gentamicin, Sigma-Aldrich (St. Louis, MO) and 0.05μg/mL amphotericin B, Sigma-Aldrich (St. Louis, MO). All cells were grown at 37⁰C in an incubator with 5% CO_2_.

### Minpp1 sequence collection and analysis

The cDNA sequence of *hminpp1 variant-1* was retrieved from the public domain of the NCBI database. To identify the spliced variants of *hminpp1*, the full-length sequence of *hminpp1 variant-1* (NCBI RefSeq: NM_004897.5, 1464 bp) was integrated into an online analytical tool ⸻Basic Local Alignment Sequence Tool (BLAST) (https://blast.ncbi.nlm.nih.gov/Blast.cgi)⸻ to search across the NCBI curated RefSeq records for related sequences with a percent identity close to 100%. Multiple sequence alignment of *hminpp1 variants* was performed using the CLUSTAL O (1.2.4) online tool.

A discrepancy between the two databases (UniProt and NCBI) was observed regarding the number of hMinpp1 isoforms and their sequences. UniProt’s database reports four-hMinpp1 isoforms, as opposed to three isoforms in the NCBI database. Therefore, as a foundation for future studies, this study is limited to the NCBI database. Multiple amino acids (AAs) sequence alignment of hMinpp1 isoforms was performed using the CLUSTAL O (1.2.4) analytical tool.

### Phylogenetic tree analysis

Minpp1 isoform-2 AAs sequence was extracted, and a BLASTP search was done through the NCBI curated refseq_protein records for related sequences across species. A distance tree of BLASTP results was constructed for hMinpp1 isoform-2. The "fast minimum evolution" method was used with a minimum sequence difference of 0.85 to construct the phylogenetic tree based on the Girishin (protein) model. The percent identity of sequences ranged from 79% to 100%.

### Quantitative real-time PCR

Treated and untreated cells were harvested for RNA isolation using Quick-RNA Kits, Zymo Research (Irvine, CA), and 1μg of RNA was reverse transcribed into cDNA using Super-script III, Invitrogen (Waltham, MA), as per manufacturer’s protocol. An iCycler iQ Multicolor Real-Time PCR detection system, Bio-Rad laboratories (Hercules, CA) was used to perform qRT-PCR on diluted cDNA with SYBR Green PCR master mix, Thermo Fisher Scientific (Waltham, MA). The housekeeping gene (18S) was amplified using primers: 5’-AGGCCGCGGAAGTTTGAGGC-3’ and 5’-ATCAGTGTAGCGCGCGTGGG -3’. *hminpp1-1* (NM_004897.5) variant was amplified using sense primer: 5’-CGGACGTCGCAGATATGGAG-3’ and anti-sense primer: 5’-TGCAGCTGGATCGACTGTT-3’. *hminpp1-2* (NM_001178117.2) was amplified using sense primer: 5’- AAATGCAGGTCTCAGCCAAT-3’ and anti-sense primer: 5’-GGGGTTCCTTGTCTTTGAAG-3’. Samples were analyzed using the ’comparative count’ method with 18S as described in the iCycler manual, Bio-Rad laboratories (Hercules, CA). At least three independent biological experiments were performed for each cDNA and its primers, and for each biological replicate, three technical replicates were run simultaneously.

### Plasmid transfection and confocal immunofluorescence microscopy

MCF-7 cells were plated at a density of 5x10^4^ on glass coverslips in a 24-well plate. After 24h of incubation, cells were transfected with human *minpp1 variant-1&-2* gene expression plasmids using TransIT-BrCa Transfection reagent, Mirius Bio (Madison, WI). Briefly, 50μL OptiMem media, Thermo Fisher Scientific (Waltham, MA), 0.5μL (1μg/μL) plasmid DNA, and 1.5μL TransIT-2020 reagent was sequentially mixed and incubated for 30min at room temperature (RT). This mixer was added dropwise to different areas to cover the well uniformly. Cells were incubated for 48-72h at 37°C and 5% CO_2_. Minpp1 expression was observed under an Olympus IX71 fluorescence microscope for positively transfected cells.

MCF-7 breast cancer cells were fixed and permeabilized before immunostaining as described by Scheffler and colleagues [52] with modifications. Briefly, positively transfected cells were washed and fixed in 3.7% cold formaldehyde solution for 10min at RT. Next, Permeabilization and blocking solution (0.1% Saponin, 1% BSA, and 0.3M Glycine) was added to the cells and kept for 30min at RT. Cells were later probed with primary antibodies (Mouse anti-CD63 mAb (H5C6-DSHB) (Iowa City, IA), rabbit anti-Minpp1 polyclonal antibody, Fabgennix Inc. (Frisco, Texas), mouse anti-LAMP2 mAb (H4B4-DSHB) (Iowa City, IA), mouse anti-GRP-78 mAb (E-4), Santa Cruz Biotechnology (Dallas, Texas) diluted at 1:200 in blocking buffer for 1h at RT. Next, cells were washed thrice with PBS and labeled with goat anti-mouse Alexa Fluor-555, goat anti-mouse Alexa Fluor 647, goat anti-mouse Alexa Fluor 488, and goat anti-rabbit Alexa Fluor 555 each at 1:400 dilution, Santa Cruz Biotechnology (Dallas, Texas). Finally, cells were mounted using mounting media containing DAPI (300nM) for nuclear staining. Double immunofluorescence images were observed under Zeiss LSM 880 laser confocal microscope, and the Zeiss Zen 2.3 software was used to analyze them.

To quantify the co-localization of proteins in the cell, Mander’s Overlap Coefficient (MOC) was employed to analyze the data mathematically [53]. First, MOC, tM1 and tM2 values were gathered and averaged (n>5) for each positively transfected and immunostained cell for determining colocalization. Then, the co-localized puncta were selected and analyzed using the Coloc2 algorithm in Fiji-ImageJ software with the Costes’ threshold regression. The reported co-occurrence of fluorescence among pixels was above the threshold to avoid background noise [53]. MOC ranged between 0 and 1; the greater the number, the more substantial evidence of the co-localization.

### Isolation of extracellular vesicles-exosomes

**Fig 1:**
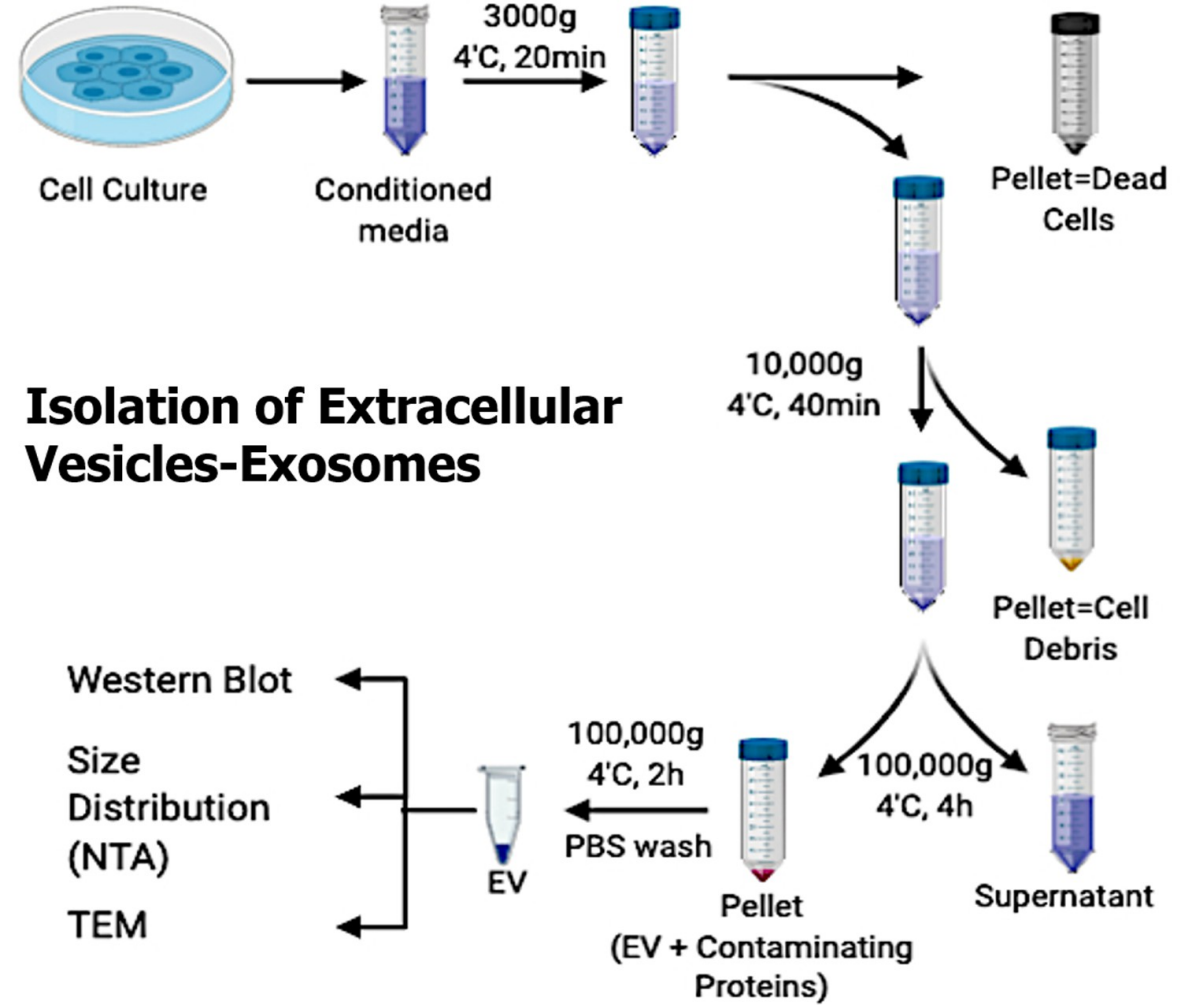
Isolation of exosomes: Schematic representation for the isolation procedure of exosomes from cell-conditioned media.

Exosomes were isolated from MCF-7 cells as described using differential centrifugation as described by Théry et al. 2006 [54] with modifications. Briefly, conditioned media treated or untreated were pooled and centrifuged at 3,000g for 20min, followed by centrifugation at 10,000g for 35min at 4°C to remove any cell debris. The clear supernatant was then ultra-centrifuged at 100,000g for 4h at 4°C using fixed-angle rotors (F50L-8 x 39). Finally, the pellet containing exosomes was washed with PBS by ultra-centrifugation again for 2h at 4°C to remove any contaminating proteins. The pellet (exosomes) formed was re-dispersed in PBS for electron microscopy and western blot (WB) analysis. For enzymatic analysis, an enzyme assay buffer was used to resuspend the pellet (Fig 1).

### Brefeldin A treatment

To study the effect of BFA on exosomes and exosomes-derived Minpp1 isoform-2, we isolated exosomes from BFA-treated MCF-7 breast cancer cells as described by McCready et al. [55]. Briefly, cells were treated overnight with 10 μg/mL BFA, Sigma Aldrich (St. Louis, MO) or vehicle as control. Conditioned media was collected and pooled for sequential centrifugation as described above for the isolation of exosomes. Finally, isolated exosomes were resuspended in PBS for further analysis.

### Western blotting

Samples for western blot analysis were prepared as described by Kilaparty [56]. Briefly, trypsinized cells were PBS washed and suspended in 1x RIPA buffer, EMD Millipore (Burlington, MA) containing Halt protease inhibitor cocktail, Thermo Fisher Scientific (Waltham, MA) and lysed for 30 min on ice. Then, cell lysates were centrifuged at 11,000g for 30min, 4°C. Finally, the pellets were discarded, and the supernatants were used immediately or stored frozen at -80°C till further use. Protein quantification was done using Bradford colorimetric assay [57] using BSA, Sigma Aldrich (St. Louis, MO), as a standard.

SDS-PAGE and western blotting (WB) were performed as described by Agarwal et al. [58]. Briefly, aliquots of 35–40μg of cell lysate protein or 2–5μg of exosome protein were boiled for 5min with 1x Laemmlli buffer, Bio-Rad Laboratories (Hercules, CA) containing 10% beta-mercaptoethanol. Proteins were then separated by 12% SDS-PAGE. Resolved proteins were electrophoretically transferred onto a nitrocellulose membrane, Bio-Rad Laboratories (Hercules, CA). The membranes were blocked for 45 min at RT with 5% non-fat dry milk prepared in Tris-Buffer Saline (20mm Tris-HCl and 150mM NaCl, pH 7.4) containing 0.01% tween-20 (TBST). The membranes were probed with primary antibodies (mouse anti-CD63 mAb (H5C6-DSHB) (Iowa City, IA), rabbit anti-minpp1 polyclonal antibody, Fabgennix Inc. (Frisco, Texas), mouse anti-GFP tagged mAb, Protein Tech Group (Rosemont, IL), and mouse anti-β-Actin antibody, Santa Cruz Biotechnology (Dallas, TX) each diluted at 1:1000 in blocking buffer for overnight at 4°C. The membranes were washed three times with TBST. The binding of primary antibodies was proved by incubation with appropriately diluted horseradish peroxidase (HRP)-conjugated secondary antibodies for 1h at RT in blocking solution. The blots were washed three times with TBST and developed with super signal west pico plus chemiluminescent reagent, Thermo Fisher Scientific (Waltham, MA) followed by autoradiography.

### Perturbation of vesicle integrity

Microsomes and exosomes are known to have a sturdy lipid bilayer than their parent cell’s lipid bilayer. Therefore, the perturbation of vesicles was performed as described by Ali et al. [19] with certain modifications. Briefly, CHAPS (3-((3-cholamidopropyl) dimethylammonio)-1-propanesulfonate), a non-denaturing mild detergent, can exclude any interference without much compromising the functional activity or integrity of enzymes. For exosomes, CHAPS has a minimum effect on their morphology or biomarker distribution [32]. In this study, we used Minpp1 enzymatic assay buffer (50mM Bis-Tris, pH-6.1, 100mM KCl, 1mM EDTA, 0.5mM EGTA, 0.05% (w/v) BSA and 3mM CHAPS) as described by Ali et al. [19] to open up microsomal vesicles to fully expressed luminal Minpp1 enzymatic activity. The Minpp1 enzymatic buffer to perturb exosomes was similar to that of microsomal vesicles, except CHAPS was increased to 16mM [32].

### Minpp1 enzyme assay

#### By enzyme linked-immunosorbent assay

Minpp1 enzyme utilizes several inositol phosphates, including Ins(1,3,4,5)P_4_ and Ins(1,2,3,4,5,6)P_6_ as substrates and removes the 3-phosphate group from the inositol ring. Samples for Minpp1 assays were prepared as described previously [19] with certain modifications. Briefly, exosomes and microsomes were resuspended in Minpp1 enzymatic assay buffer and incubated with substrate ⸻Ins(1,3,4,5)P_4_. Samples were then subjected to Ins(1,4,5)P_3_ detection using an ELISA kit, My BioSource (San Diego, CA), following manufacturers’ instructions. Briefly, enzyme preparations (exosomes and microsomes samples) were pre-incubated with 5μM Ins(1,3,4,5)P_4_ for 24h at 37 ⁰C in 50μL enzyme assay buffer. Standards and samples were then subjected to an ELISA microtiter plate already coated with goat anti-rabbit antibody at RT. Antibody specific for Ins(1,4,5)P_3_ and HRP-conjugated Ins(1,4,5)P_3_ were incubated along with samples for 1h at 37°C. After washing properly with wash buffer, an equal ratio of substrate A and substrate B was added for 1h at 37°C in the dark. The reaction was quenched later using the stop solution provided with the kit. Optical density was measured at 450nm within 10min of the reaction at 450nm using a microplate reader. The amount of Ins(1,4,5)P_3_ as a product was determined using a standard curve constructed with known amounts of Ins(1,4,5)P_3_ and run simultaneously.

#### By polyacrylamide gel electrophoresis of InsP_6_

Minpp1’s enzymatic activity in exosomes was further assessed using another substrate ⸻Ins(1,2,3,4,5,6)P_6_ (InsP_6_)⸻ as described by Wilson and his colleagues [59]. Briefly, exosomes re-dispersed in Minpp1 enzymatic assay buffer were incubated with 4.0 nmol InsP_6_ for 24h at 37 ⁰C followed by extraction and analysis of the products using Poly-Acrylamide Gel Electrophoresis (PAGE). Briefly, 33% polyacrylamide/TBE gels were used to resolve inositol polyphosphates present in the samples. The stacking gel had the composition of 0.2mL of 80% acrylamide/ bisacrylamide (19:1), 0.2mL of TBE (10x), 10μL of 10% APS, 3μL of TEMED and dH_2_O to a total volume of 2mL. The mounted gel was pre-run at 100V/10mA for 20min. About 30μL of InsP_6_ standards (1-16nmol of InsP_6_ (dipotassium salt from Sigma Aldrich (St. Louis, MO)) and exosome samples were mixed with TriTrack DNA Loading Dye (6x), Thermo Fisher Scientific (Waltham, MA) before loading unto mounted gel. The gel was run at 100V/10mA till the orange dye reached 2/3^rd^ of the distance. The resolved gel was stained at RT with toluidine blue staining solution (20% methanol, 2% glycerol, and 0.05% toluidine blue) at RT until the bands showed up. The gel image was later captured, and band density was determined using the ImageJ software to semi-quantitate the bands.

### Nanoparticle tracking analysis

Exosomes’ particle size was determined by Nanoparticle Tracking Analysis (NTA) using ZetaView PMX120. Briefly, the purified exosomes were diluted at 1:50–1:5000 in PBS and subjected to NTA (ZetaView PMX120). Then, the particle numbers and size were graphed for the average size of the isolated exosomes.

### Transmission electron microscopy

Transmission Electron Microscopy (TEM) was performed to observe the size and heterogeneity of exosomes. Briefly, 5μL aliquots of treated and untreated exosomes were applied to a 300 mesh Formvar/carbon-coated grid, Electron Microscopy Sciences (Hatfield, PA) and left for 5min at RT. After that, the excess solution was removed by filter paper, and the samples were left to dry. The samples were later negatively stained with 1% uranyl acetate (UA) and observed under the FEI Tecnai F20 electron microscope. Two independent experiments were performed, and several photographs were taken for each experimental condition.

### Statistical analysis

The qPCR data were statistically analyzed using One-Way ANOVA (Analysis of Variance) with post-hoc Tukey HSD (Honestly significant difference) using SAS software. All experiments were repeated at least three times independently, and each sample was run in triplicate.

## Results

### Bioinformatics’ alignment of hMinpp1 variants and isoforms

#### Sequence analysis

**Fig 2:**
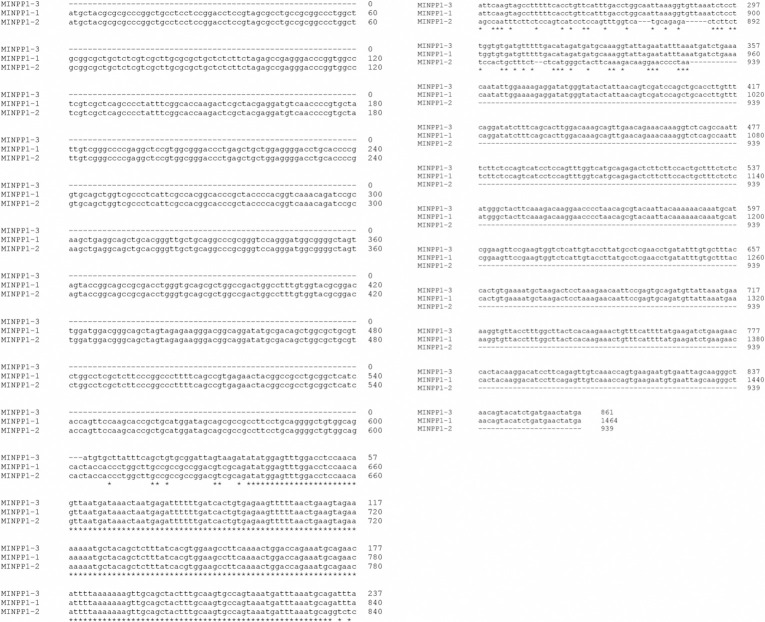
Nucleotide sequence alignment of multiple variants of *hminpp1*: Three variants of the *hminpp1* gene are identified. These *variants* are formed by alternative splicing of the gene. Homology modeling of the full-length sequences of the three *variants* collected from the NCBI database were aligned to evaluate sequence similarity. *hminpp1 variant1* (NM_004897.5) is the full-length gene (MINPP1-1). *hminpp1 variant-2* (MINPP1-2) comprises similar 5’ sequence but lacks substantial 3’ sequence. *hminpp1 variant-3* (MINPP1-3) lacks 5’ sequence but retains 3’ sequence.

To study heterogeneity in Minpp1 protein’s function and relate their physiological significance, nucleotide sequences available in databases were first analyzed to identify the spliced *hminpp1 variants* and their transcriptional products. To find related sequences across taxonomic species, we initially BLASTN was initially performed on the full-length parent sequence: hminpp1 *variant-1*, NCBI Refseq: NM_004897.5, 1464 bp. In total, two more unique variants were found in humans with a percent identity close to 100% (Fig 2).

It is presumably due to the splicing of *hminpp1 variant-1*: a) it truncates two exons to yield *variant-2* (NCBI Refseq: NM_001178117.2) with 104 unique base pairs of a total of 939 bp, b) it truncates an additional exon yielding 861 bp *hminpp1 variant-3* (NCBI Refseq: NM_001178118.2), the shortest variant amongst them, with an unaligned 34 bp 5’ region.

A discrepancy has been observed between databases ⸻UniProt and NCBI⸻ regarding the number of hMinpp1 isoforms reported. Previously, this lab reported four hMinpp1 isoforms based on NCBI and Uniport databases. However, with renewed interest in analyzing current hMinpp1 sequences using the updated NCBI database, the presence of only three hMinpp1 isoforms could be affirmed. As opposed to the NCBI, UniProt still reports one extra Minpp1 variant/protein. Therefore, as a foundation to imminent research, this investigation is limited our investigation to the NCBI database.

**Fig 3:**
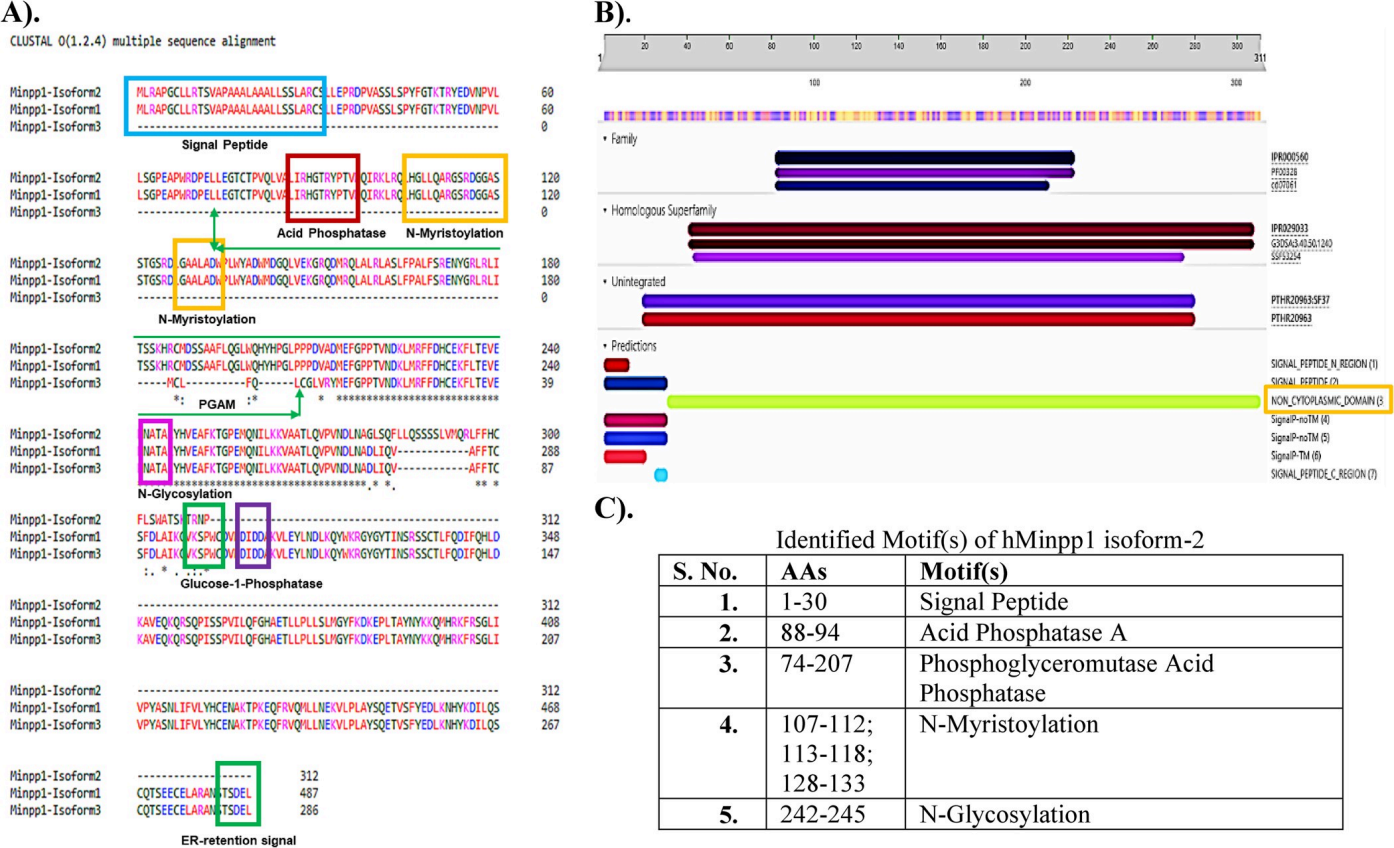
Minpp1 isoform-2 amino acid sequence analysis. **A)**. Multiple AAs sequence alignment of isoforms of hMinpp1. Three isoforms of hMinpp1 were identified as a translated outcome of the alternatively spliced *hminpp1* gene. Homology modeling of the entire AAs sequences of three isoforms collected from NCBI sequence aligned to evaluate sequence similarity. AAs 213–278 were relatively conserved for all isoforms of hMinpp1. Isoform-1 (NP_004888.2) is a full-length protein. Isoform-2 (NP_001171588.1) comprises similar N-terminal sequences but lacks C-terminal sequences. Isoform-3 (NP_001171589.1) lacks the majority of N-terminal sequences but retains C-terminal sequences. The "NATA": N-Glycosylation site is conserved in all three isoforms, while glucose 1-phosphatase (DIDD) and ER retention (SDEL) motifs were absent in isoform-2, **B).** The InterPro protein viewer for the Minpp1-isoform-2 (NP_001171588.1). The underlying tool—InterProScan—annotates protein’s domain and motifs using predictive models provided by multiple databases [60], **C).** Summary of the prospective motifs in hMinpp1 isoform-2 based on its sequence alignment with hMinpp1 isoform-1.

This lab’s previously published work reported various motifs in the parent protein, hMinpp1 isoform-1 (NP_004888.2) [22]. Therefore, upon aligning hMinpp1 sequences of all three isoforms, this study analyzed whether the motifs are shared in all three hMinpp1 isoforms (Fig 3A).

Interestingly, we found only one hMinpp1 isoform-1 signature motif (amino acids) common in all three isoforms ⸻N-glycosylation (NATA, AAs 242–245). However, the bioactivity of the N-glycosylation motif is not yet analyzed. Therefore, since the primary focus of this study is on hMinpp1 isoform-2, all the prospective motifs/domains found in hMinpp1 isoform-2 (NP_001171588.1) are summarized in Fig 3C.

### hMinpp1 isoforms alignment

In Minpp1 isoform-2, the phosphoglyceromutase acid phosphatase (PGAM) domain spans across AAs 74–207. Within the PGAM domain, N-Myristoylation and Acid Phosphatase A (AP-A) motifs were shared between hMinpp1 isoform-1 and -2 but not isoform-3. The AP-A motif (AAs 88–94; RHGTRYP) is known to be highly conserved across species and is well-known for dephosphorylating inositol polyphosphates (InsPs) [25]. The presence of the myristoyl group increases protein to lipid interaction altering its subcellular localization [41–44, 61]. Therefore, the absence of an ER-retention signal (KDEL) and the presence of the N-Myristoylation motif in hMinpp1 isoform-2 implies its extra-ER presence. As an alternate approach, InterProScan was used, an analytical tool to predict protein domains and motifs by scanning multiple databases [60], to examine the hMinpp1 isoform-2 amino acids sequence. This annotated a non-cytoplasmic domain (Fig 3B) with different motifs.

**Fig 4:**
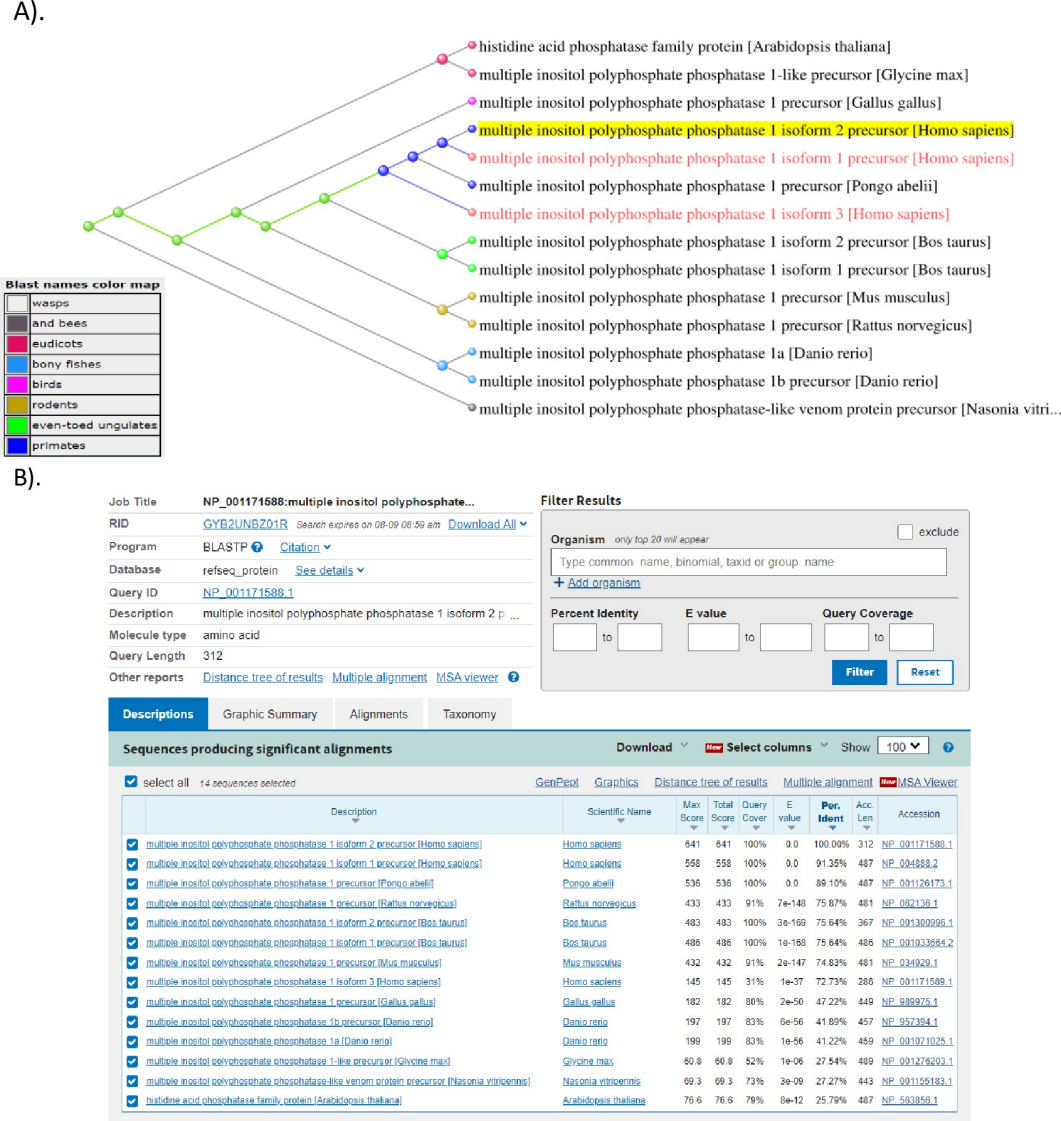
hMinpp1 isoform-2 (NP_001171588.1) (highlighted yellow) related amino acid sequences across species **A).** A rooted slanted cladogram **B).** BLASTP was used to compute the hMinpp1 isoform-2 amino acid sequence in a pair-wise alignment against the curated NCBI refseq_protein database to construct the above-mentioned distance-based cladogram. The higher pairing sequences were included in the tree, displaying homology across taxonomic species—percent identity ranged: 25.79% to 100%. The search was limited to records that exclude models (XM/XP). hMinpp1 isoform-1: NP_004888.2. hMinpp1 isoform-3: NP_001171589.1.

A rooted phylogenetic tree was constructed to study the distribution of hMinpp1 isoform-2 related proteins across taxonomic species and establish its evolutionary relatedness and divergence. The cladogram (Fig 4A) reflects hMinpp1 isoform-2 related proteins across taxa. An evolutionary pattern reveals the conservation of hMinpp1 isoform-2 protein across species such as bats, whales, dolphins, even/odd-toed ungulates, rabbits and hares, primates, rodents, carnivores, placental, etc., with sequence percent identity between 25.79% to 100%. The NCBI refseq_protein records were used to extract the sequences and construct a distance-based dendrogram, hence the reliability and reproducibility of the tree. Phylogenetic analysis suggests a close relation between hMinpp1 isoform-1 and isoform-2 (Fig 4).

### ER stress affects the relative expression of *hminpp1 variants*

**Fig 5:**
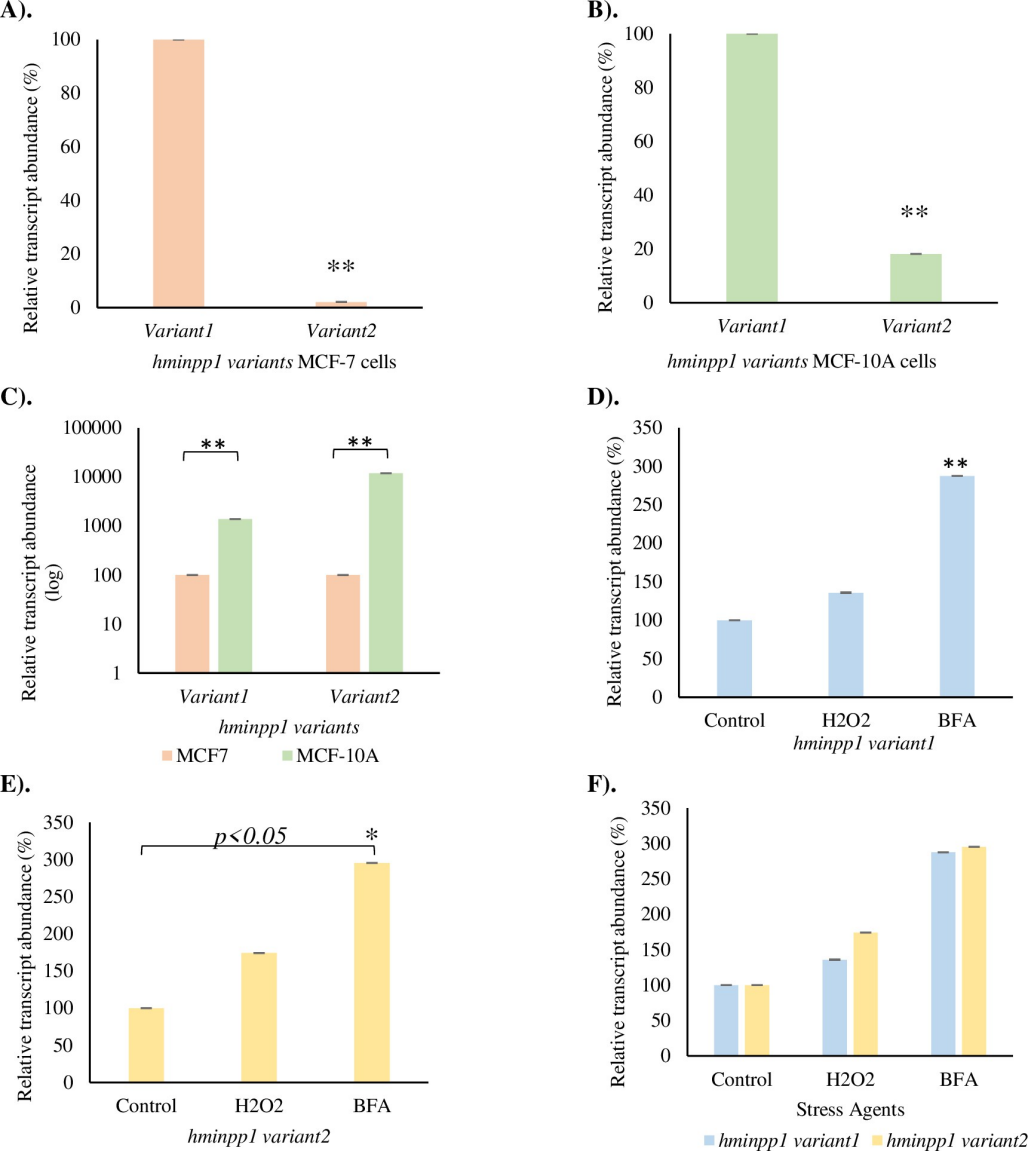
Expression profile of *hminpp1 transcript variants* by real-time PCR in **A).** MCF-7 breast cancer cells. **B).** MCF-10A normal breast cells. **C).** a comparative expression analysis of *hminpp1 variants*. *hminpp1 variants* expression is normalized to 100% in MCF-7 cells to better analyze each variant’s difference compared to MCF-10A cells. **D).** *hminpp1 variant1* expression in MCF-7 cells under different stress conditions. **E).** *hminpp1 variant2* expression in MCF-7 cells under different stress conditions, (p<0.05) **F).** Comparative expression analysis of *hminpp1 variant-1 and -2* under cellular stress. *hminpp1 variant-1* expression is normalized to 100% in the control sample to analyze the relative difference in each variant better. Data, except in C, were converted into a percentage of control for better representation. Hydrogen peroxide (H_2_O_2_): 100μM; BFA: 10.0μg/mL. Data reported as mean values of mRNA abundance after normalization with 18S gene expression. The error bars represent the standard error of the mean (±SEM). One-way ANOVA with post-hoc Tukey HSD (Honestly Significant Difference) was used to measure the significance between groups with significance *p<0*.*01 (A*, *B*, *C*&, *D)* and *p<0*.*05*(E).

Earlier work from this lab suggested Minpp1 protein as an ER stress responder, the expression of which was enhanced under stress conditions [56]. However, whether the stress-induced expression is limited to hMinpp1 isoform-1 or is consistent with isoform-2 is unknown. Due to the unavailability of hMinpp1 isoform-specific antibodies, this study examined the relative transcript abundance of *hminpp1 variants-1 and -2* in MCF-7 breast cancer and its non-cancer MCF-10A cells under different stress conditions using real-time PCR with gene-specific primers. It is found that the relative transcript abundance of *hminpp1 variant-1* was considerably higher than the *hminpp1 variant-2* in both cell lines (Fig 5A–5C). Inducing cellular stress increases the relative expression of each variant to a similar extent, suggesting both *hminpp1 variant-1&-2* genes respond to cell stress in a similar proportion (Fig 5D–5F). These results suggest that not only hMinpp1 isoform-1 but also isoform-2 plays a role in cellular stress responses.

### Minpp1 isoform-2 is localized in extra-ER vesicles

**Fig 6:**
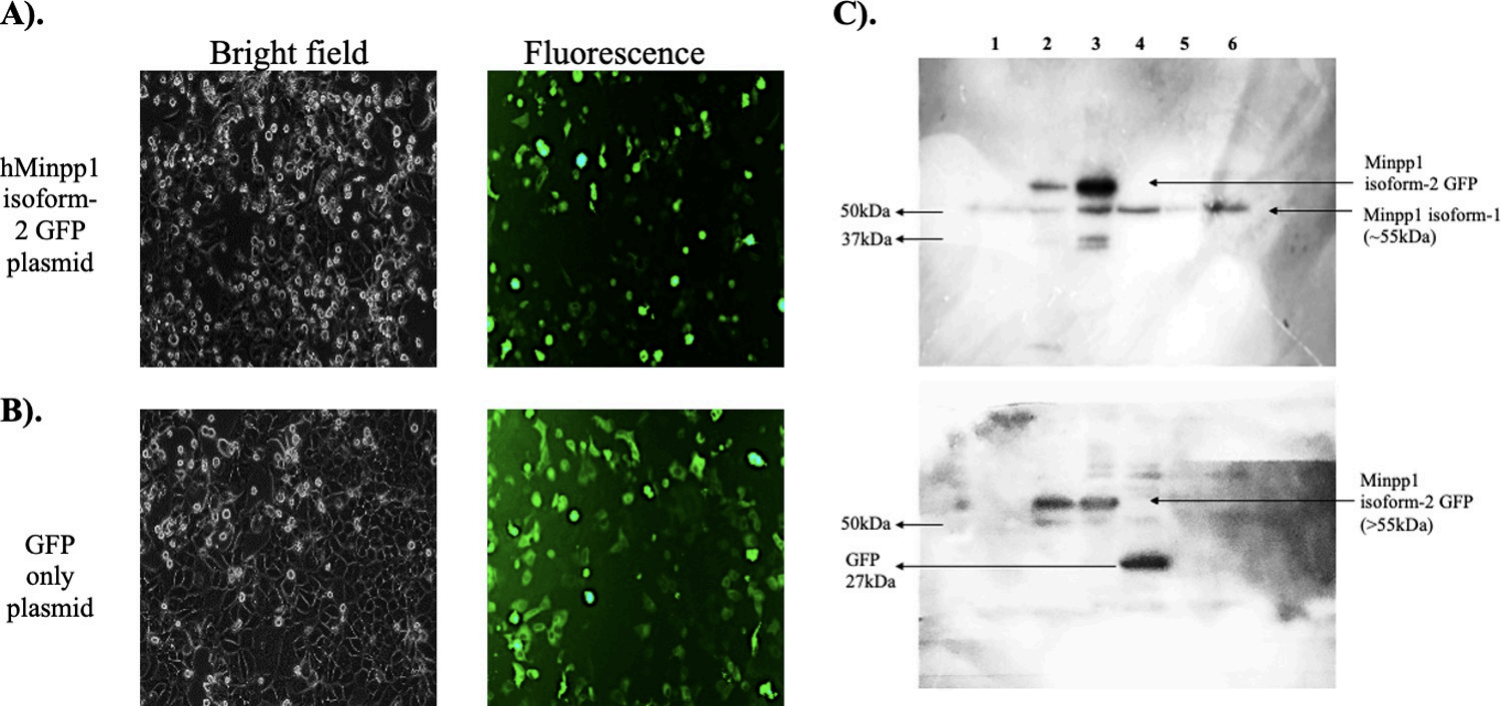
Plasmid Expression analysis: Bright field and fluorescence images of MCF-7 breast cancer cells successfully transfected and incubated 24h with **A).** Minpp1 isoform-2 plasmid (GFP tagged), **B).** GFP only plasmid. **C).** Positively transfected cells were lysed, and SDS-PAGE resolved proteins were transferred onto nitrocellulose membrane to further immuno-stain with anti-Minpp1 polyclonal antibody (upper blot) and anti-GFP monoclonal antibody (lower blot). Results show that the polyclonal antibody raised against Minpp1 isoform-1 also shared its epitope with Minpp1 isoform-2. GFP MW: 27kDa. Lane-1, 5 & 6: Control cell lysate (CL), Lane-2 & 3: Minpp1 isoform-2 GFP CL, Lane-4: GFP alone CL.

Since Minpp1 isoform-2 lacks ER retention signal and is expressed at a relatively low level, GFP-tagged Minpp1 isoform-2 was transiently overexpressed using the expression plasmid to study its subcellular localization relative to the known ER location of Minpp1 isoform-1. Undoubtedly, overexpressing GFP-tagged Minpp1 isoform-2 will also help study any extra-ER secretory pathway. Therefore, MCF-7 cells transfected with hMinpp1 isoform-2 expression plasmid were also characterized for GFP tagged hMinpp1 protein using fluorescence microscopy and Western blot analysis. The presence of green fluorescence in Fig 6A confirms the expression of hMinpp1 isoform-2 and GFP proteins. Furthermore, western blot analysis employing an anti-Minpp1 antibody (Fig 6C, upper blot) that shares common epitopes and thus recognize both Minpp1 isoform-1 and -2, and anti-GFP antibody (Fig 6C, lower blot) further confirms the expression of GFP-tagged hMinnp1 isoform-2 protein (Fig 6C). It is because of the low-level expression of Minpp1 isoform-2 and its extracellular secretion, its endogenous expression is missing as compared to Minpp1 isoform-1. Additionally, Minpp1 isoform-2 specific antibody is not yet available.

**Fig 7:**
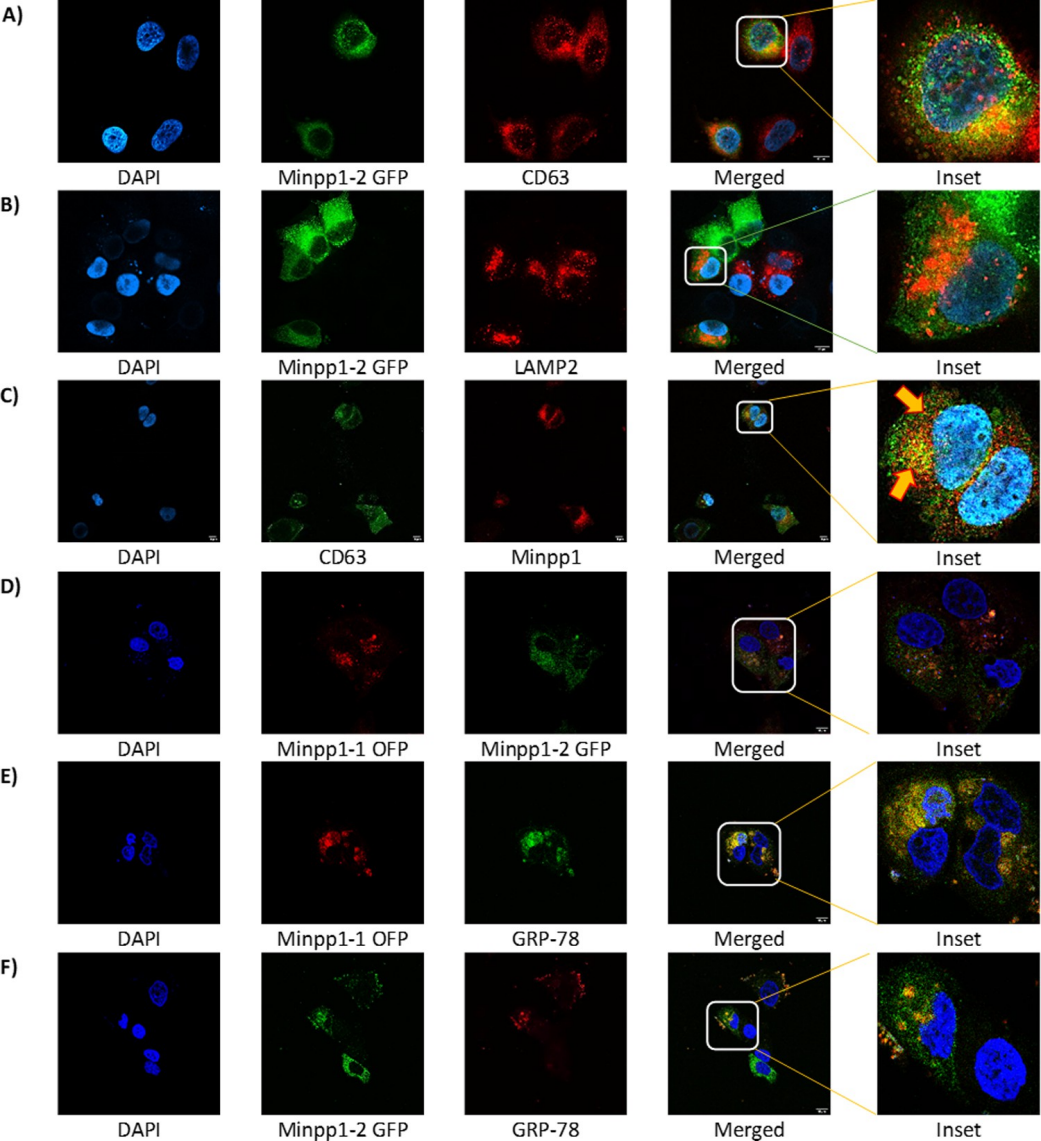
Confocal immunofluorescence image analysis on 4% paraformaldehyde (PFA) fixed MCF-7 cells: **A).** hMinpp1 isoform-2 GFP transfected MCF-7 cells were immuno-stained with Mouse anti-CD63 antibody (MVB/exosomes biomarker). **B).** hMinpp1 isoform-2 GFP transfected MCF-7 cells were immunostained with mouse anti-LAMP2 antibody (Lysosomal biomarker). **C).** Cells were immunostained with Rabbit anti-Minpp1 and Mouse anti-CD63 antibody (MVB/exosomes biomarker). **D).** Minpp1 isoform-1 OFPSpark and Minpp1 isoform-2 GFP transfected MCF-7 cells. **E&F).** Minpp1 isoform-1 OFPSpark and Minpp1 isoform-2 GFP transfected MCF-7 cells were stained with mouse anti-GRP-78 antibody (ER biomarker). Secondary antibody staining was done by goat anti-mouse Alexa Fluor 555 (Red); Goat anti-mouse Alexa flour 488 (Green); Goat anti-mouse Alexa Fluor 647 (far Red); and goat anti-rabbit Alexa flour 555 (Red). Nuclei were stained with DAPI (blue). Scale bar is 10μm.

A pronounced secretion of Minpp1 in extra-ER compartments [33, 34, 51] raised concern due to its inherent ER retention signal [21]. However, an isoform ambiguity surrounds the reported Minpp1 protein. The collective findings in this study led to hypothesize that Minpp1 isoform-2 has an extra-ER localization, perhaps into lysosomes and exosomes. If this hypothesis turns out true, it would also then mean that the Minpp1 enzymatic activity observed earlier in tumor cell culture media could well be due to Minpp1 isoform-2 [51]. Therefore, studies were initiated to investigate the localization of the Minpp1 isoforms. Minpp1 isoform-1 OFPSpark and Minpp1 isoform-2 GFP plasmids were transfected in MCF-7 cells. Transfected and non-transfected MCF-7 cells were immunostained with anti-CD63, anti-GRP-78, anti-Minpp1 polyclonal, and anti-LAMP2 antibodies followed by confocal immunofluorescence microscopy (Fig 7). Consistent with the hypothesis, Minpp1 isoform-2 was found in the lysosomes (Fig 7B) and multivesicular bodies (MVBs) (Fig 7A), colocalizing with organelle-specific biomarkers LAMP2 (Manders’ tM1 (Above auto threshold of Ch2 (LAMP-2)), 0.303; Manders’ tM2 (Above auto threshold of Ch1 (Minpp1 isoform-2 GFP)), 0.561) and CD63 (Manders’ tM1 (Above auto threshold of Ch2 (CD63)) 0.253; Manders’ tM2 (Above auto threshold of Ch1 (Minpp1 isoform-2 GFP)) 0.536, respectively. In Fig 7C, a co-localization (superimposed yellow color) can carefully be observed in particular regions of non-transfected cells (Manders’ tM1 Above auto-threshold of Ch2 (CD63)) 0.449; Manders’ tM2 (Above auto-threshold of Ch1 (Minpp1)) 0.343), symbolizing Minpp1 isoform-2 enveloped in MVBs. The isolated/scattered red dots around the periphery of the nucleus, perhaps ER, represents an ER-localized Minpp1 isoform-1 (Fig 7C).

In Fig 7E & 7F, Minpp1 isoform-1 OFPSpark cells (Manders’ tM1 Above auto-threshold of Ch2 (GRP-78)) 0.629; Manders’ tM2 (Above auto-threshold of Ch1 (Minpp1-OFP)) 0.570) was found relatively more localized with anti-GRP-78 than Minpp1 isoform-2 GFP (Manders’ tM1 Above auto-threshold of Ch2 (GRP-78)) 0.295; Manders’ tM2 Above auto-threshold of Ch1 (Minpp1 isoform-2 GFP)) 0.294). Furthermore, co-transfection of both Minpp1 isoform-1 OFPSpark and Minpp1 isoform-2 GFP exhibited a rare co-localization (Manders’ tM1 Above auto-threshold of Ch2 (Minpp1 isoform-2 GFP)) 0.022; Manders’ tM2 Above auto-threshold of Ch1 (Minpp1-OFP)) 0.0314) (Fig 7D).

### Minpp1 isoform-2 is localized in exosomes

**Fig 8:**
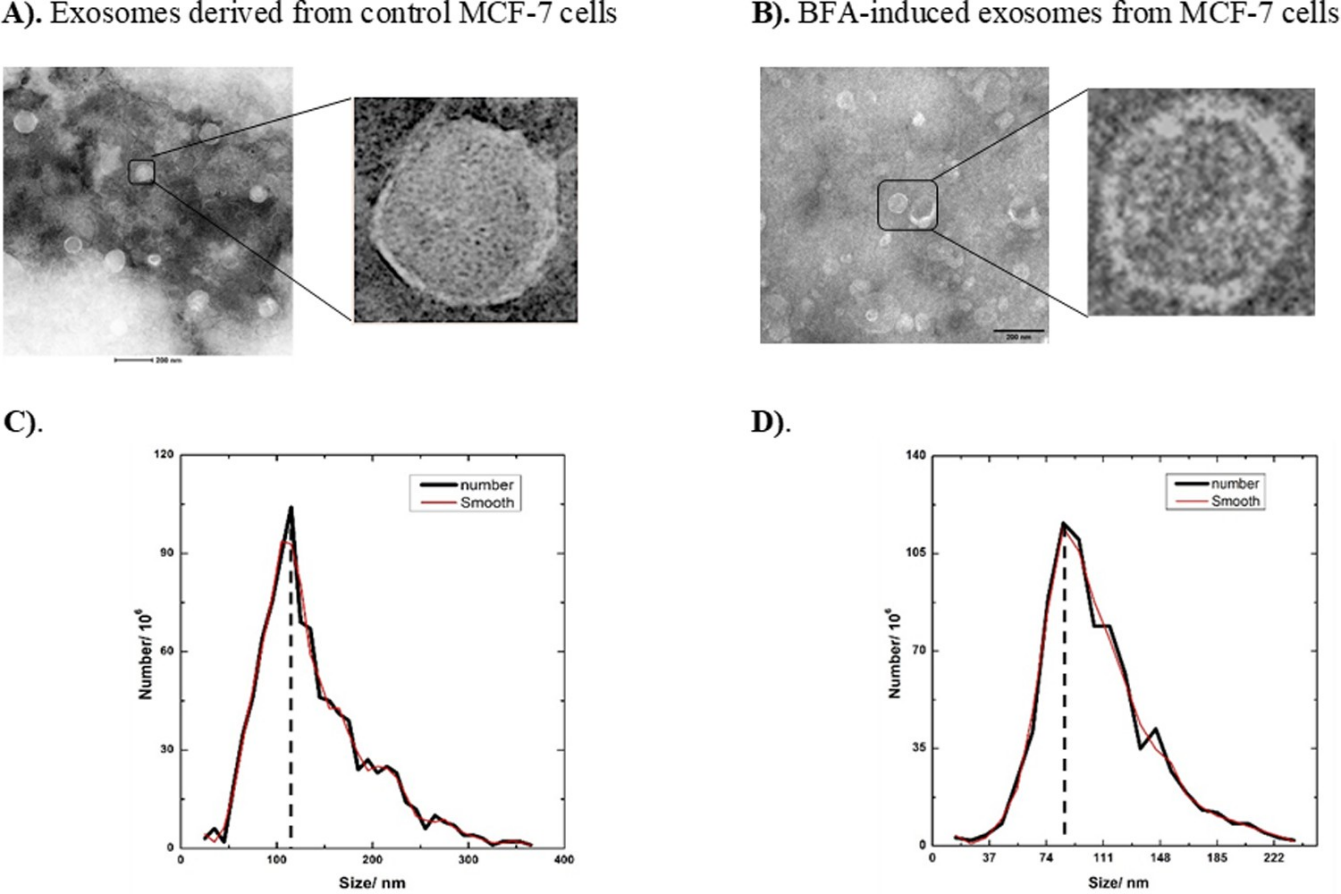
Characterization of exosomes: TEM micrographs of heterogeneous exosomes isolated from A). Control MCF-7 cells, and B). MCF-7 cells treated with BFA (10μg/mL) inhibitor for 24hr. Scale bar: 200nm C). Nanoparticle tracking analysis of isolated exosomes. The distribution peaks around ~120 nm, following the exosome enrichment during preparation. D). An uninterrupted secretion of smaller exosomes, distribution peaks around ~95 nm, were reported in the presence of a BFA (10μg/mL) inhibitor for 24hr. 1% Uranyl Acetate for 15sec at RT was used to stain the samples negatively.

Sorting into MVBs could deliver proteins to both lysosomes and extracellular space. Next, we examined the extracellular secretion prospect of the Minpp1 isoform-2 was examined by identifying its presence in exosomes. Isolated exosomes, maintained for 24h in the serum-free media of MCF-7 cells, were isolated and characterized using western blotting, Transmission Electron Microscopy (TEM), and Nanoparticle Tracking Analysis (NTA). TEM images of isolated exosomes exhibited a cup-shaped double membrane morphology (Fig 8A) with an average size of 120nm (Fig 8C), typical for exosomes.

**Fig 9:**
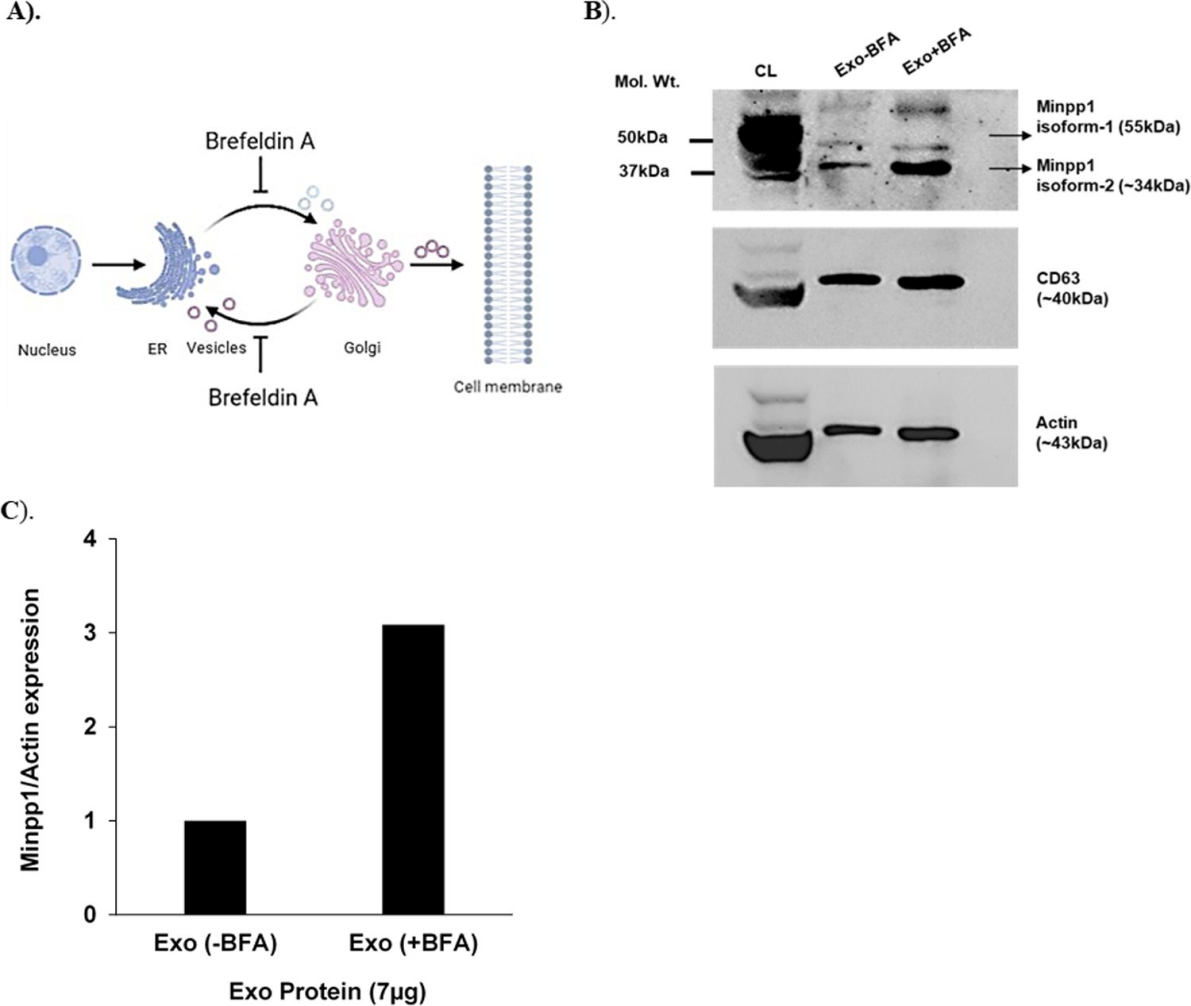
Analysis of Minpp1 isoform-2 expression in BFA induced MCF-7 exosomes: **A)**. Mechanistic illustration of protein trafficking inhibitor-BFA, **B)**. Western blot analysis of exosomes (Exo) isolated from BFA (10μg/mL, 24h) treated and untreated MCF-7 cells conditioned media, pre-enriched by sequential centrifugation. An equal amount of proteins (7μg) from both control and BFA-treated Exo were loaded. Probing was done by; anti-Minpp1 antibody (Fabgennix Inc.) that also binds to Minpp1 isoform-2, anti-Exo biomarker: CD63 antibody (Santa Cruz Biotech Inc.) and, β-Actin antibody (Santa Cruz Biotech Inc.) at 1:1000 dilution in blocking buffer, CL; cell lysate (40μg protein). Shown blots are a representative result of three independent experiments (n = 3). **C)**. A comparative analysis of Minpp1’s expression percentage) between BFA treated and untreated Exo sample. A more than 3-fold increase in Minpp1 isoform-2 secretion in EVs from BFA treated cells was observed.

The western blot analysis exhibited enrichment of CD63, a known biomarker for exosomes, in the isolated fractions (Fig 9B), further confirming their identity. Note that there is a greater degree of fluctuation in CD63 band densities in total cell lysates and exosomes. This is perhaps due to the difference in the amounts of proteins loaded in cell lysates (40μg) and exosomes (7 μg). Immunostaining of the blot with anti-Minpp1 polyclonal antibody confirmed the presence of Minpp1 isoform-2 in exosomes (Fig 9B). Note that this antibody shares the common epitopes in Minpp1 isoform-1 (55kDa) and isoform-2 (34kDa) and thus stains both.

A cell produces/releases multiple types/sizes of vesicles as EVs [62]. Therefore, it was investigated whether an interruption of the vesicular trafficking pathway would affect exosome secretion and incorporation of Minpp1 isoform-2 in it. A known pharmacological protein trafficking inhibitor, brefeldin A (BFA) [63], was used to analyze the extra-ER secretion of Minpp1 isoform-2 into exosomes. Initially, the difference in size and morphology in the exosomes isolated after BFA treatment were examined (Fig 8). Then the packaging of Minpp1 isoform-2 in BFA-induced exosomes was assessed.

This study found an uninterrupted secretion of exosomes in the presence of BFA. However, a noticeable reduction in the size of exosomes was observed (Fig 8D). Briefly, exosomes derived from untreated cells displayed an average size of 120nm compared to 95nm for exosomes isolated from BFA-treated cells (Fig 8C & 8D). In contrast, no significant morphological difference was recorded between exosomes isolated from treated or untreated cells (Fig 8A & 8B). Both sources of exosomes exhibited a typical double-membrane cup-shaped morphology.

### ER-stress increases the secretion of Minpp1 isoform-2 in exosomes

Next, it was determined whether the presence of Minpp1 isoform-2 and exosome’s primary biomarker CD63 are altered under BFA-induced cellular stress. BFA is known to inhibit intracellular vesicular trafficking and cause cellular stress (Fig 9A).

The data revealed an irrepressible extracellular secretion of Minpp1 (Fig 9B) and CD63 in the isolated exosomal fraction. It can be speculated that cancer cells envelop and release more of the Minpp1 isoform-2 enzyme into extracellular space during cellular stress conditions. A semi-quantification of the western blot bands accounted for more than a two-fold increase in the expression of Minpp1 isoform-2 in the BFA-treated exosomes compared to control exosomes (Fig 9C). Note that the densities of actin band in total cell lysates and exosome samples vary drastically due to differences in the amounts of proteins loaded in cell lysate (40 ug) and exosomes (7ug). However, it is comparable between control and BFA-treated exosomes.

### Minpp1 isoform-2 in exosomes is enzymatically active

Next, it was investigated whether Minpp1 isoform-2 present in exosomes is enzymatically active, Minpp1 isoform-2, similar to isoform-1, as proposed to have an AP-A motif, as found in our amino acid sequence alignment (Fig 3A). The conserved InsPs 3-phosphatase activity was examined using two different techniques: ELISA and PAGE.

**Fig 10:**
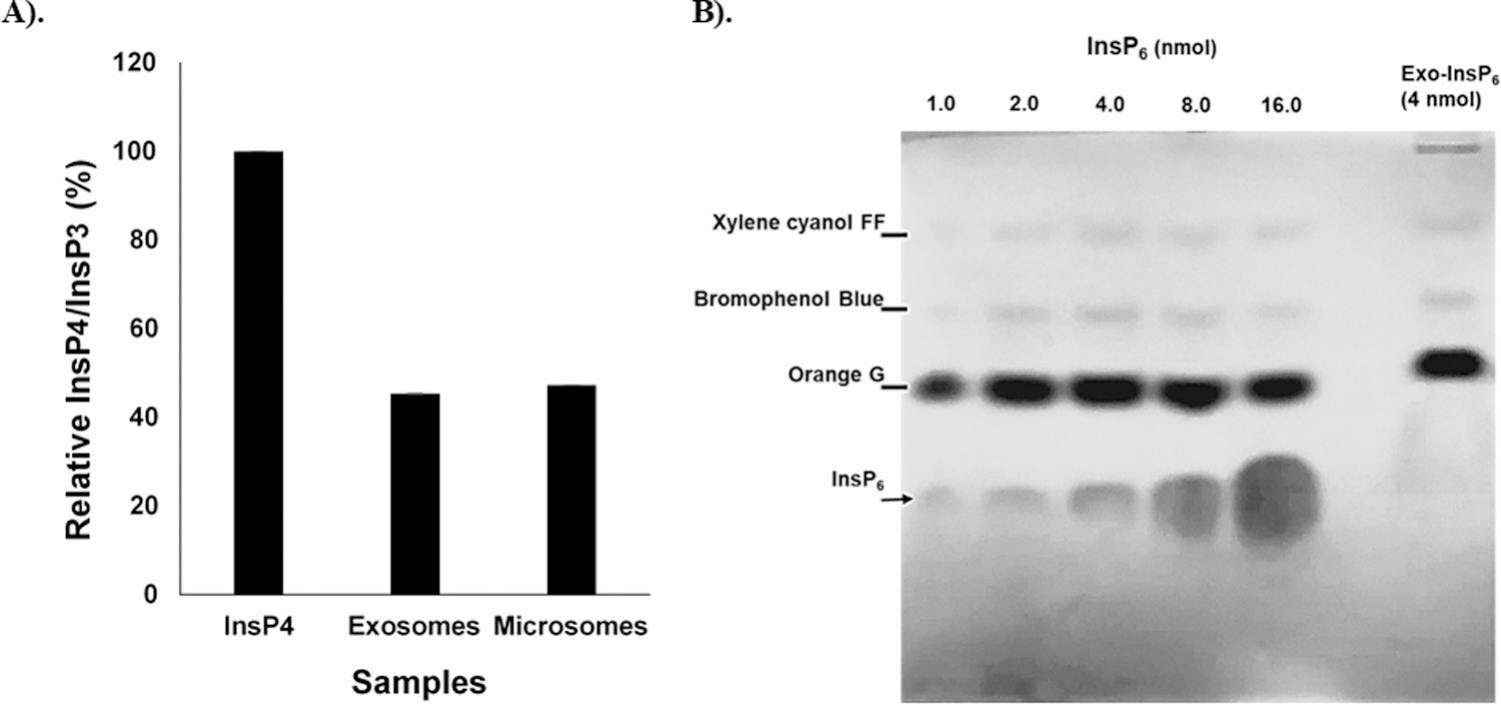
Enzymatic analysis of exosome-based Minpp1 isoform-2. **A).** Competitive inhibition enzyme immunoassay against exosomes pre-enriched by ultra-centrifugation. The ELISA plate was pre-coated with goat anti-rabbit antibody. Samples were subjected to the plate along with antibodies specific to InsP_3_ and Horseradish Peroxidase (HRP)-conjugated InsP_3_. The competitive inhibition reaction is launched between HRP labeled InsP_3_ and unlabeled InsP_3_ with the antibody. On adding HRP substrate solution, color develops reciprocally to the amount of InsP_3_ in the sample. Samples were spiked with 5μM InsP_4_ for 24h RT. Exosomes (~1.0μg) were resuspended in Minpp1 assay Buffer with 16mM CHAPS. Microsomes (~1.0μg) were resuspended in Minpp1 assay Buffer with 3mM CHAPS. The bar graph shows the percentage of InsP_4_ dephosphorylation as a qualitative activity of Minpp1 isoform-2 enzyme compared to control (InsP_4_+Minpp1 Assay Buffer). **B).** Hydrolysis of InsP_6_ (4 nmol) with exosomes as an indication of Minpp1 isoform-2 enzyme activity. Freshly collected serum-free conditioned media was differentially ultra-centrifuged to isolate exosomes. The isolated exosomes were resuspended in Minpp1 enzymatic assay buffer and later incubated overnight with InsP_6_ (4 nmol) at room temperature. After incubation, metabolized InsPs were resolved by PAGE and visualized with toluidine blue staining.

In a competitive inhibition ELISA assay, Ins(1,3,4,5)P_4_ (InsP_4_) was used as a substrate which is known to be dephosphorylated to Ins(1,4,5)P_3_ (InsP_3_) by Minpp1. The product InsP_3_ was then detected by ELISA kit colorimetrically employing specific antibodies to InsP_3_. We found about a 60% reduction in the ratio of InsP_4_/InsP_3_ concentration in exosomal fraction and microsomes (resuspended in Minpp1 enzymatic buffer) compared to control (spiked with InsP_4_) (Fig 10A). Microsomes were used as a positive control known to carry the Minpp1 isoform-1 enzyme.

The enzymatic activity of Minpp1 isoform-2 in exosomes was also determined qualitatively by hydrolysis of Ins(1,2,3,4,5,6)P_6_ as a substrate because Minpp1 is known to hydrolyze multiple InsPs including InsP_6_. Following incubation of InsP_6_ (4nmol) with exosomes, the metabolic products were separated on PAGE to analyze InsP_6_ dephosphorylation. No InsP_6_ band was found in the presence of exosomes, while toluidine staining qualitatively detected InsP_6_ standards ran in parallel with other controls (S1 Fig). In conclusion, InsP_4_ and InsP_6_ dephosphorylation by exosomes confirm the bioactivity of exosomes-enveloped Minpp1 isoform-2 enzyme.

## Discussion

This study examined the heterogeneity in Minpp1 and demonstrated an extra-ER Minpp1 isoform-2 secreted in exosomes. Computational studies previously published from this lab predicted four different isoforms of Minpp1 [22] in humans. However, this study re-evaluates existing databases and consolidates to only three variants of the *hminpp1 gene* (NM_004897.5) translated into three different isoforms (Fig 3). We found that the difference was due to an obsolete NCBI’s RefSeq: XM_017016966.1 (*hminpp1 variant-4*), which no longer retains its "validated" status in the database. Irrespective of the number of isoforms, the exact mechanism behind isoform multiplicity, however, remains unclear. It could be due to some unknown evolutionary mechanism that increases the Minpp1(NP_004888.2) proteome diversity, linking different motifs or domains into the isoforms (Fig 3C), thus altering their cellular localization and function.

Among the noted motifs and domains, hMinpp1 isoform-2 was found to be the only other member of the Minpp1 family retaining the evolutionarily conserved acid phosphatase (AP-A) motif known to hydrolyze InsPs with no ER-retention signal (KDEL). Minpp1 isoform-2 was also found to have N-Myristoylation and N-Glycosylation sites in the linear amino sequence of Minpp1 isoform-2 (Fig 3C). However, the functionality of any motif/domain of the Minpp1 isoform-2 protein has not yet been biochemically determined. Bearing that in mind, it is predicted that Minpp1 isoform-2 could escape the ER by anchoring the lipid membrane via its N-Myristoylation site, possibly into an extra-ER compartment. The non-cytoplasmic domain found in the “InterProScan” of the Minpp1 isoform-2 sequence additionally supports the prediction (Fig 3B).

The BLASTP-phylogenetic tree constructed based on hMinpp1 isoform-2 amino acid sequence showed that the related proteins are widely spread from eudicots (flowering plants), bony fishes, rodents, avians to humans (Fig 4A). Such protein sequence identity-based phylogenetic tree could identify functional similarities [64]. However, considering how nature exploits a protein’s structure based on its environment, examining the BLASTP-phylogenetic tree alone on enzymes’ functional transferability [65] will not be sufficient. An advanced computational approach targeting functional residues or protein’s structural/surface properties could uncover more on Minpp1 isoform multiplicity, functional constraints, and the protein divergence across taxa.

The bioinformatics approach is limited to answering whether these isoforms are functional? When expressed, do they contribute to a new function to the hMinpp1 proteome or play a regulatory role? Therefore, an experimental approach is imperative. This study now presents data indicating that *hminpp1 variant-2* is expressed in real-time in MCF-7 cancer cells, albeit the expression levels are insignificant compared to *hminpp1 variant-1*. However, during cellular stress conditions, a significant increase in the relative expression of the normally almost dormant *hminpp1 variant-2* was observed. The low-level expression might represent a defense mechanism where a cell responds to stress by inducing specific genes essential for stabilizing the stress environment. The *hminpp1 variant-2* could also adhere to the *hminpp1 variant-1* convention as a potential cell-stress responder [56].

**Fig 11:**
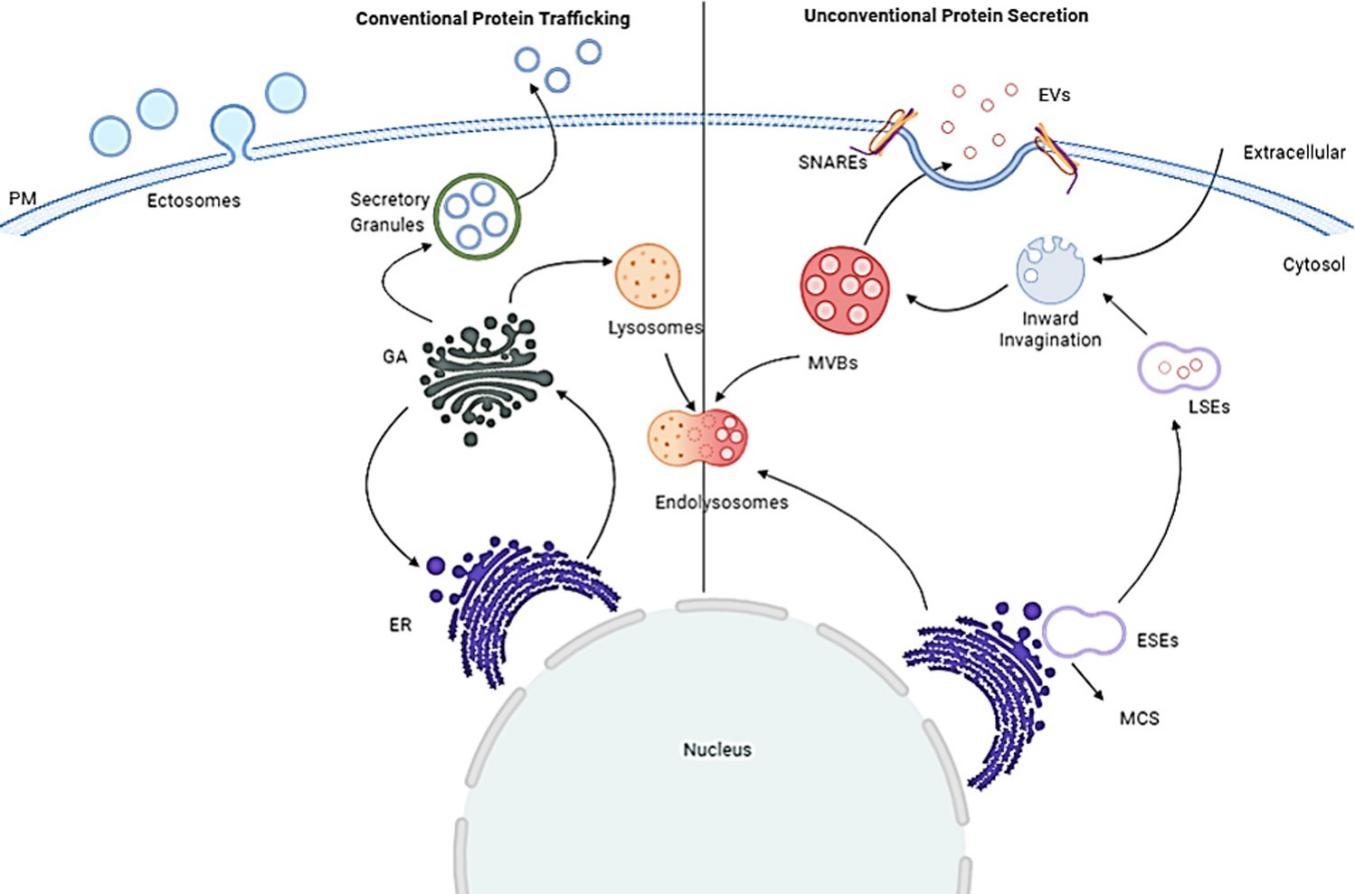
Pictorial depiction of protein secretory pathway: Conventional vs. Unconventional protein pathway.

Lately, several researchers have reported the presence of the Minpp1 enzyme in extra-ER compartments [32–36, 51]. However, it is unclear which isoform of Minpp1 protein it is. Minpp1’s presence outside ER is beyond the paradigm of conventional protein secretion due to the ER-retention signal in isoform-1. Therefore, the proteome diversity of Minpp1 could help understand its extra-ER presence and segregation away from its cytosolic physiological substrate, InsPs [19]. This study established that the two isoforms of Minpp1 partially reside together in the same vicinity (Fig 7D). Minpp1 isoform-1 was found relatively more localized around the nucleus, with ER biomarker (GRP-78) than Minpp1 isoform-2 (Fig 7C, 7D & 7F). Minpp1 isoform-2 was more on the cell periphery, the vicinity of PM-destined MVBs (Fig 7C, 7D & 7F), and was found colocalized with MVB biomarker CD63 (Fig 7A). In the UPS pathway, MVBs can release their cargo into an intermediate endo-lysosomal compartment (Fig 11) for degradation or fuse with PM to release the contained ILVs into the extracellular space [66]. With the presence of Minpp1 isoform-2 in the lysosome (Fig 7B), one could argue that its secretion is due to the unconventional secretory (UPS) pathway [37, 44, 67, 68]. It is further interesting to speculate that the enzyme reported in the lysosome and extracellular cell culture media in Windhorst’s study [51] could well be Minpp1 isoform-2. However, the significance of Minpp1 in the hostile environment of lysosomes is still unclear.

The PM-destined MVBs contain signalosomes [43] or ILVs (future exosomes) implicated in various immune modulations and programmed cell death-based diseases [69]. Endocytic generated mixed population of ILVs [68] has come a long way, from garbage bags [70] to therapeutic agents [71], enclosing a diverse mixture of RNAs, miRNA, proteins, lipids, and glycans [46]. This study presents the first experimental evidence that signalosomes in MCF-7 cells carry Minpp1 isoform-2 (Fig 7B) before discharging its content into extracellular space, enveloped in exosomes; hence, the extracellular presence of Minpp1 isoform-2.

The ER stress-related apoptotic pathways have extensively been targeted in various diseases, including neurodegenerative disorders [72], cancer, type-2 diabetes [73], intestinal inflammation [74], amyotrophic sclerosis (ALS) [75], and many more. Some of these disorders such as neurodegenerative [76, 77], single nucleotide polymorphisms (SNP) mutations that affect the milk fatty acids (FAs) traits in Chinese Holstein [78], a glycolytic bypass in Hepatitis-B virus (HBV) positive hepatocellular carcinoma (HCC) [79], differentiation and apoptosis [51, 80] are implicated in involving Minpp1. Previous work from this group has reported a link between Minpp1 and ER stress [56]. In this study, an equivalent increment in the relative transcript abundance of the *minpp1 variant-2* gene further suggests a similar functional causality with ER stress (Fig 5). Severe ER stress releases exosomes carrying damage-associated molecular patterns (DAMPs) [81]. The reported exosomes from tumor cells remodel the ECM by regulating the pre-metastatic niche [82]. These DAMPs-associated exosomes carry a heterogeneous group of molecules, including ATP, uric acid, smaller and larger proteins [81], and factors for self-renewal and protection [83]. There is an irrepressible secretion of relatively smaller exosomes (~95nm) during ER-stress by BFA (Fig 8C & 8D) accompanied by a concomitant increase in the expression of exosomes-associated Minpp1 isoform-2 enzyme (Fig 9B & 9C). However, only a limited amount of unconventionally secreted proteins are resilient enough to surpass the inhibitory effect of BFA, i.e., independent of ER/Golgi apparatus (Fig 11) [37, 84]. Collectively, these findings support the hypothesis that an unconventional protein secretion pathway ushers Minpp1 isoform-2 into extracellular space. Also, the overexpression of Minpp1 isoform-2 in ER stress-exosomes could perhaps be a cell-stress alarmin. Moreover, since exosomes suggest parental imprint [46], the presence of Minpp1 isoform-2 in exosome-associated DAMPs could be viewed as a promising biofluid-based non-invasive early breast cancer biomarker.

The data presented in this study further report that the proposed AP-A motif is enzymatically active in the exosomes-associated Minpp1 isoform-2 (Fig 10). Thus, packaging enzymatically active Minpp1 isoform-2 into exosomes could facilitate the exogenous transfer of Minpp1 isoform-2 from one cell to another or assist in ECM remodeling by protecting tumor cells against the anti-proliferative actions of any extracellular InsPs [85, 86]. Moreover, the Minpp1’s ability to remove 3-phosphate overlaps with PTEN [20]. Therefore, and as suggested for PTEN’s expression in the tumor’s microenvironment [87], the expression of Minpp1 isoform-2 enzyme in exosomes could imply an essential role in the evolution of ECM and tumor cells during metastasis. It is further interesting to speculate that the Minpp1 isoform-2 enzyme in ECM could activate a cassette of proteins in its proximity that collectively function in metastasis and evading cell death. Thus, inhibition of the Minpp1 isoform-2 enzyme could inhibit ECM’s protective potential, making Minpp1 isoform-2 an attractive target for drug therapy to restrict tumor invasion.

## Supporting information

S1 Fig **A).** InsP_3_-ELISA logarithmic standard curve. The best fit curve was plotted with the InsP_3_ concentration log on the x-axis vs. the OD log on the y-axis. Regression analysis was used to analyze the graph. **B).** varying concentrations of BSA spiked with 4 nmol of InsP_6_ were resolved by PAGE to examine any effect of added protein on the detection of InsP_6_.
(TIF)
