# Supplementary figures and images for: Identification and functional characterization of multiple inositol polyphosphate phosphatase1 (Minpp1) isoform-2 in exosomes with potential to modulate tumor microenvironment
Source: PLoS One. 2022 Mar 2;17(3):e0264451. doi: 10.1371/journal.pone.0264451 (PMC8890658; doi:10.1371/journal.pone.0264451)

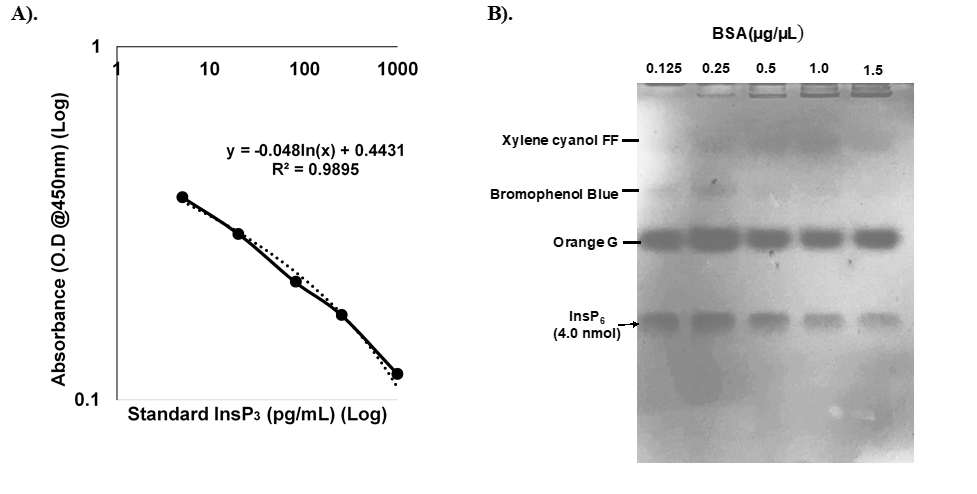

Supplement: S1 Fig — A). InsP3-ELISA logarithmic standard curve. The best fit curve was plotted with the InsP3 concentration log on the x-axis vs. the OD log on the y-axis. Regression analysis was used to analyze the graph. B). varying concentrations of BSA spiked with 4 nmol of InsP6 were resolved by PAGE to examine any effect of added protein on the detection of InsP6. (TIF) [file pone.0264451.s001.tif]
